# Longitudinal Association Between Child Psychological Abuse and Neglect and Academic Achievement in Chinese Primary School Children: A Moderated Mediation Model

**DOI:** 10.3389/fpsyg.2022.870371

**Published:** 2022-04-25

**Authors:** Jiajing Li, Ziying Li, Xiuya Lei, Jingyuan Yang, Xiao Yu, Haoning Liu

**Affiliations:** Department of Psychology, School of Humanities and Social Sciences, Beijing Forestry University, Beijing, China

**Keywords:** academic achievement, Chinese primary school children, learning engagement, family SES, child psychological abuse and neglect

## Abstract

To investigate the relationships among child psychological abuse and neglect (CPAN), children’s learning engagement, family socioeconomic status (family SES), and children’s academic achievement, 271 children (*M*_age_ = 9.41 ± 0.81 years old) and their parents participated in this study with a longitudinal design. Results revealed that learning engagement at T1 mediated the relationship between CPAN at T1 and academic achievement at T2 when gender, age, grade, and academic achievement at T1 were under control. Family SES at T1 moderated the relationship between children’s learning engagement at T1 and academic achievement at T2. The association between learning engagement and academic achievement was stronger among children from lower family SES. Our findings highlighted the negative impact of CPAN and the critical role of learning engagement in children’s academic achievement, especially for those from low SES families.

## Introduction

Child psychological abuse and neglect (hereafter, CPAN) has been a serious public health and social concern in the West (U.S. Department of Health and Human Services et al., 2020), Eastern Europe (Sebre et al., 2004), and Asia (Haque et al., 2021; Yu et al., 2021). The case is similar in China where the prevalence of child abuse was about 37% (Ji and Finkelhor, 2015) and that of neglect of 13 year old was around 49% (Cui et al., 2016). Although it has been widely demonstrated that CPAN could increase the risk of poor academic achievement during childhood (Nikulina et al., 2011; Raby et al., 2019; Scharpf et al., 2021), the roles of individual factors (i.e., children’s learning engagement) and family factors (i.e., family socioeconomic status and hereafter, family SES) in its mechanism have not been identified yet. Clarifying how CPAN contributes to children’s academic achievement can provide theoretical insight into the adverse factors of children’s academic development and may help to guide the intervention with children who have experienced CPAN in practice.

Recent evidence demonstrated that CPAN was inversely correlated to children’s academic achievement (McGuire and Jackson, 2018; Zhao et al., 2021), which was related to a crucial individual factor—learning engagement (Mullins and Panlilio, 2021). Specifically, a higher level of CPAN decreased children’s learning engagement, and thus, it impeded academic achievement of children. However, there were only samples from the United States to support the mediation mechanism of learning engagement underlying CPAN and children’s academic achievement (Mullins and Panlilio, 2021). Other countries, like China, gave no empirical support. In traditional Chinese culture, especially Confucianism, parents always regard child psychological abuse (e.g., harsh rebuke) as loving and caring (Qiao and Chan, 2005). One of the reasons is that they resort to abuse to improve children’s learning engagement, in order to improve the child’s academic performance. This is different from western culture (Liao et al., 2011). As a result, it is important to explore the relationship among CPAN, learning engagement, and academic achievement in China. In addition, recent studies show that the link between children’s learning engagement and their academic achievement may be influenced by family factors, such as family SES (Tucker-Drob and Harden, 2012; Lawson and Farah, 2017; Chen et al., 2021). Thus, this study aimed to investigate whether and how CPAN contributed to children’s academic achievement through their learning engagement and to examine the moderation of family SES in Chinese primary school children.

### Child Psychological Abuse and Neglect and Academic Achievement

CPAN, also known as child psychological maltreatment, refers to continuously and repetitively inappropriate parenting practices and can be characterized by five types: threatening, ignoring, belittling, intermeddling, and corrupting (Pan et al., 2010). Specifically, abuse involves parents using words and expressions to threaten or humiliate children, restricting them, or encouraging their inappropriate behavior. However, it does not involve physical or sexual contact. Neglect means parents’ chronic neglect of children’s needs (Pan et al., 2010; Liu F. et al., 2020). Compared with other forms of abuse and neglect, child psychological abuse and neglect is less likely to be identified and addressed (Baker and Brassard, 2019; Baker et al., 2021). In this study, the term of psychological abuse and neglect referred to that children imposed by parents.

Studies highlighted that CPAN and children’s academic achievement were significantly and negatively correlated (De Bellis et al., 2013; Romano et al., 2015; McGuire and Jackson, 2018; Su et al., 2019; Strathearn et al., 2020). For example, De Bellis et al. (2013) found that children from 6 to 18 who had been abused and neglected were worse off in mathematics and reading than those who had never been. Meta-analysis evidence (McGuire and Jackson, 2018) also demonstrated that children with experiences of abuse and neglect had lower academic achievement than those without. Recently, a 21-year longitudinal study with 5,200 children highlighted the negative relationship between CPAN and long-term educational outcomes (Strathearn et al., 2020).

However, other studies demonstrated such relationship was insignificant (Eckenrode et al., 1993; Perez and Widom, 1994; Briscoe-Smith and Hinshaw, 2006; Tanaka et al., 2015). For instance, researchers found no significant differences between abused and non-abused ADHD girls in mathematics and reading (Briscoe-Smith and Hinshaw, 2006). Tanaka et al. (2015) also found that those child victims of sexual abuse had similar teacher-rated school performance as those who were not. However, it is worth noting that the participants in the former study were children with atypical development (children with attention deficit hyperactivity disorder), whereas in the latter study, the children’s academic achievement was only evaluated by their teacher. More evidence is required from normal children by standard academic tests. Furthermore, researchers have also noted that the relationship between CPAN and children’s academic achievement may be affected by other factors (Todd and Wolpin, 2003), such as family factors (i.e., family SES) and individual ones (i.e., learning engagement). Therefore, it is essential to investigate what mechanisms and conditions of CPAN affect children’s academic achievement to account for these inconsistent results.

### Learning Engagement as a Mediator

As a motivational outcome, learning engagement is concerned with participation in initiating and learning activities (Gonida et al., 2009; Skinner et al., 2009; Xiong et al., 2021), which manifests within the behavioral, emotional, and cognitive components (Archambault and Dupéré, 2017; Olivier et al., 2021). Behavioral engagement involves efforts and persistence in learning activities (Xiong et al., 2021). Emotional engagement includes feelings about school, such as a sense of belonging to the school as well as connection with teachers and peers (Pears et al., 2013; Lam et al., 2014). Cognitive engagement emphasizes on cognitive strategies such as focusing attention (Corno and Mandinach, 1983).

According to ecosystem theory (Bronfenbrenner, 1979; Krishnakumar and Black, 2002; Romano et al., 2015; McGuire and Jackson, 2018), children’s development is influenced by interlocking nested environments. Specifically, distal family factors (i.e., CPAN) may influence their developmental outcomes (i.e., academic achievement) through proximal individual factors (i.e., learning engagement; Shonk and Cicchetti, 2001; Mullins and Panlilio, 2021). In other words, children’s learning engagement may mediate between CPAN and their academic achievement.

In addition, empirical studies have indicated how children’s learning engagement mediated the relationship between CPAN and their academic achievement. Firstly, the significant and negative relationship between CPAN and children’s learning engagement has been found (Font and Maguire-Jack, 2013; Johnson and Sinatra, 2013; Pears et al., 2013; Ringle et al., 2020). For example, Pears et al. (2013) found that abused and neglected children had lower emotional and cognitive engagement than other children. Ringle et al. (2020) also noted that CPAN was directly related to poor learning engagement in a longitudinal study. Secondly, children’s learning engagement and academic achievement were positively and significantly correlated (Fredricks et al., 2016; Leonard et al., 2016; Hershberger and Jones, 2018; Ahun et al., 2020; Zhao et al., 2021). For instance, Leonard et al. (2016) identified that learning engagement could positively predict children’s reading and mathematics performance. Ahun et al. (2020) also demonstrated that cognitive and behavioral engagement significantly predicted children’s later academic achievement. Based on the convergent evidence, it is reasonable to assert that children’s learning engagement might mediate between CPAN and academic achievement.

### Family SES as a Moderator

Family socioeconomic status (Family SES) broadly represents the ranking or accumulated capital of an individual or a family in a socio-cultural system (Hackman et al., 2010; Chen et al., 2021; Coetzee et al., 2021). Transactional theory (Sameroff, 2009) has emphasized that children’s development is influenced by the interaction between the individual and his or her environment. It has also been shown that family SES played a role when learning engagement predicts children’s academic achievement (Tucker-Drob and Harden, 2012). Therefore, family SES may moderate between children’s learning engagement and academic achievement.

In China, parents’ parenting beliefs about children’s educational attainment vary according to family SES (Chi and Rao, 2003). As a Chinese saying goes, “knowledge changes destiny,” suggesting that low SES families strongly believe that children’s learning engagement can enhance academic achievement. Parents and children from low SES families believe that only by studying hard and improving their academic achievement can children change their future (Chi and Rao, 2003; Behrman et al., 2017). Furthermore, these families do not have sufficient capital (e.g., educational resources) to meet the needs of children so that children can only improve their academic achievement on their own, such as through more learning engagement (Kim and Fong, 2013). Thus, the relationship between children’s learning engagement and academic achievement was more prominent for children from low SES families (Shah et al., 2018). On the other hand, in high SES families, parents emphasize on holistic development and create an artistic atmosphere, cultivating parent–child reading habits, etc. (Niklas et al., 2020; Yuan et al., 2021). Meanwhile, parents in high SES families help their children promote learning skills; thus, learning engagement is not the only way to improve academic achievement. In summary, in China, family SES may moderate the relationship between learning engagement and academic achievement. The correlation between learning engagement and academic achievement is stronger for children from low SES families than those from high SES families.

### The Current Study

**Figure 1:**
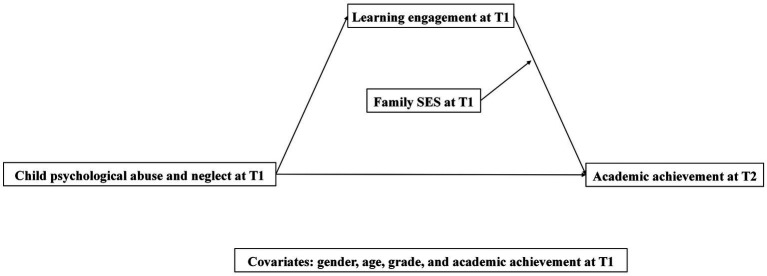
The hypothesized model of child psychological abuse and neglect, children’s learning engagement, family SES, and children’s academic achievement, controlling for gender, age, grade, and academic achievement at T1.

Although there has been some evidence of the relationships among CPAN, learning engagement, family SES, and academic achievement in cross-sectional design studies, little is known about the specific mechanism and the direction of the causality underlying such relationships (Armfield et al., 2021; Mullins and Panlilio, 2021). Thus, the main purpose of the present study was to examine the mediating role of learning engagement and moderating role of family SES underlying the relationship between CPAN and children’s academic achievement by a longitudinal design. In addition, because CPAN or learning engagement correlated more significantly to children’s academic achievement in primary school years (Chung, 2015), the study targeted elementary school children. Moreover, previous studies have indicated that children’s gender, age, and grade are statistically and significantly correlated to CPAN, learning engagement, family SES, and children’s academic achievement (Meeus, 2016; Guo et al., 2020). Thus, the current study included gender, age, and grade as covariates. The current study proposed three hypotheses (Figure 1):

*H1*: CPAN at T1 would negatively associate with children’s academic achievement at T2.

*H2*: Learning engagement at T1 would mediate the relationship between CPAN at T1 and academic achievement at T2.

*H3*: Family SES at T1 would moderate the path from learning engagement at T1 to academic achievement at T2. Specifically, the relationship between learning engagement at T1 and academic achievement at T2 is stronger for children from low family SES at T1.

## Materials and Methods

### Participants

Participants were randomly recruited from an elementary school in Shandong Province, China. There were 271 participants at T1 (146 girls; mean age was 9.41 ± 0.81 years old). Nineteen participants were ruled out due to uncompleted data in the questionnaire at T1, and three absent from the exam at T2 were also excluded. Thus, total participants at T2 were 249 (135 girls; mean age was 9.97 ± 0.69 years old). All students were typically developing children without learning difficulties. Written informed consent was received from parents.

### Measures

#### Child Psychological Abuse and Neglect

CPAN was recorded by Child Abuse and Neglect Scale (CPANS) with 23 items (Pan et al., 2010; Luo et al., 2020; Sun et al., 2020). There were five dimensions: threatening (seven items, such as *“my parents have yelled at me”*), ignoring (six items, such as *“my parents have never cared about the changes in my academic performance”*), belittling (four items, such as *“my parents have verbally abused me when I was not expecting it”*), intermeddling (four items, such as *“my parents have peeked at my diary”*), and corrupting (two items, such as *“my parents did not forbid me from drinking alcohol”*). Children gave scores ranging from 0 (never) to 4 (always) on these items. Then, the average scores were calculated, with higher scores indicating a higher level of CPAN. This measure has been widely used among Chinese students. It has been shown well reliability and well valid in previous research (Zhang and Lyu, 2017; Liu F. et al., 2020; Li et al., 2021). Confirmatory factor analyses were also conducted (CFAs) to examine the five dimensions. The CFA supported the model fit indices: *χ^2^/df* = 1.88, CFI = 0.92, TLI = 0.91, SRMR = 0.06, and RMSEA = 0.06. In the current sample, the Cronbach’s *α* of the whole measure was 0.90.

#### Learning Engagement

Children were required to indicate to what extent each statement was true for the last 6 months on a 5-point Likert scale from “1 = not at all” to “5 = fully.” Ten items were used to assess children’s learning engagement (Lam et al., 2014). For instance, *“I can study for a long time with few breaks during the period, “I feel happy when I am fully engaged in learning,” and “Learning inspires me.”* The mean scores were calculated with a higher score indicating more learning engagement. The scale has shown well reliability and validity in Chinese students (Tian and Chen, 2020; Yi et al., 2020). In the current sample, the CFA supported the model fit indices: *χ^2^/df* = 1.70, CFI = 0.98, TLI = 0.98, SRMR = 0.03, and RMSEA = 0.05. In this sample, the Cronbach’s *α* was 0.91.

#### Family Socioeconomic Status

Family Socioeconomic Status (Family SES) was measured in two aspects: caregivers’ educational background and the monthly household income (Cui et al., 2018; Zhou et al., 2018; Liu J. et al., 2020; Chen et al., 2021). The first aspect was coded with “1 = uncompleted elementary school,” “2 = elementary school graduation,” “3 = junior high school graduation,” “4 = high school or junior college,” “5 = undergraduate or college,” and “6 = master’s degree and above,” which were reversely scored to calculate the mean value of parental education. For the second aspect, a scale with 11 options (“1 = ¥0 - ¥3,999,” “2 = ¥4,000 - ¥5,999,” “3 = ¥6,000 - ¥7,999,” “4 = ¥8,000 - ¥9,999,” “5 = ¥10,000 - ¥11,999,” “6 = ¥12,000 - ¥13,999,” “7 = ¥14,000 - ¥15,999,” “8 = ¥16,000 - ¥17,999,” “9 = ¥18,000 - ¥19,999,” “10 = ¥20,000 - ¥39,999,” and “11 = upper than ¥39,999″) was used. Based on previous studies (Bradley and Corwyn, 2002), the scores of these two measurements were separately standardized and summed as an indicator of family SES, with a higher score indicating higher family SES.

#### Academic Achievement

Compared with other school subjects, Chinese and mathematics are more valued in Chinese culture. Thus, scores on these two subjects can represent students’ academic achievement at school (Ren et al., 2021; Wang et al., 2021). In this study, children’s academic achievement was assessed according to their Chinese and math scores in the final examinations based on the national compulsory education curriculum standards. Both the average Chinese and mathematics scores were obtained, and standardized by grade to get a composite academic achievement score (Sebre et al., 2004; Hibbard et al., 2012; Morrissey et al., 2014; Lv et al., 2016). Higher composite standardized scores represented better academic achievement. In this sample, Cronbach’s *α* of academic achievement at T1 was 0.77; Cronbach’s *α* of academic achievement at T2 was 0.80.

#### Covariates

Students’ gender (female and male), grade (2–4 grades), age (7–11 years old), and the composite standardized academic achievement at T1 were included as covariates in the analyses of all models.

### Procedures

Two rounds of data on children’s academic achievement were collected at school in December 2020 (T1) and June 2021 (T2). There was a semester in the six-month interval between T1 and T2. Previous studies have proven that the developmental changes in academic achievement of primary school students can be revealed in one semester (Bartee et al., 2018). Other variables were collected at T1. Children were required approximately 8 to 10 min to complete the questionnaires. In addition, it took their parents about 3 min to complete family SES survey. Before administering the test, experimenters had received rigorous and standardized training. In addition, to ensure that the participants understood the meaning of 5-point Likert scale from “1” to “5” and each item, experimenters explained the differences of the five options and each item carefully to the participants during the test. At the end of each survey, each participant received a gift as a compensation for their time. This study was authorized by the university’s ethics committee.

### Data Analysis

Firstly, preliminary analyses, including descriptive statistics and Pearson correlation analysis, were conducted by the SPSS 26.0 to provide an initial overview of the variables. Secondly, the PROCESS macro software was used to examine the mediation of learning engagement on the relationship between CPAN at T1 and children’s academic achievement at T2 (Hayes, 2013). According to Fairchild et al. (2009), the calculation formula of *R*^2^ effect size for mediation analysis is as follows: *R*^2^_med_ = *r*^2^_MY_ − (*R*^2^_Y·MX_ − *r*^2^_XY_). Thirdly, whether family SES at T1 could moderate the mediation was investigated. The moderated mediation was used to examine whether the mediation effect varies with the value of the moderator (Muller et al., 2005). The moderated mediating model was analyzed in the PROCESS macro of Hayes (2013) based on the bootstrapping method. There were 5,000 samples. The effect was significant when the confidence interval did not contain 0 (Preacher et al., 2007). Fourthly, a simple slope analysis was conducted when the moderating effect was significant. Finally, the subgroup (sensitivity) analysis among the fourth graders was conducted (the corresponding results were shown in Appendix 1).

## Results

### Preliminary and Correlation Analyses

Harman’s One-factor Test was conducted to test common method bias (Harris and Mossholder, 1996). The results showed that eigenvalues of seven factors were greater than 1 and the factor with the largest eigenvalue explained 27.34% of the variance, which was below the critical value of 40%. Therefore, there was no significant common method bias.

Table 1 presented the results of descriptive statistics (means and standard deviations) and Pearson correlations for the main variables. Specifically, CPAN at T1 was significantly and negatively correlated with learning engagement at T1 (*r* = −0.29, *p* < 0.01), family SES at T1 (*r* = −0.13, *p* < 0.05), and academic achievement at T1 (*r* = −0.18, *p* < 0.01). Learning engagement at T1 was significantly and positively correlated to academic achievement at T1 (*r* = 0.14, *p* < 0.05) and academic achievement at T2 (*r* = 0.35, *p* < 0.01). Family SES at T1 was significantly and positively related to academic achievement at T2 (*r* = 0.35, *p* < 0.01).

**Table 1:** Descriptive statistics and correlations among variables.

Variable	*M*	SD	Min.	Max.	1	2	3	4	5	6	7
1. Gender	-	-	1	2	-						
2. Grade	3.16	0.78	2	4	−0.26	-					
3. Age	9.47	0.69	7	11	−0.03	0.80^**^	-				
4. Academic achievement at T1	0.00	1.00	−4.62	1.53	−0.13^*^	0.00	−0.02	-			
5. CPAN at T1	0.66	0.50	0.00	3.22	0.01	0.17^**^	0.09	−0.19^**^	-		
6. Learning engagement at T1	3.40	1.86	1.00	5.00	−0.06	0.01	0.03	0.14^*^	−0.30^**^	-	
7. Family SES at T1	0.00	1.00	−4.77	4.55	−0.02	−0.05	−0.13^*^	0.01	−0.13^*^	0.10	-
8. Academic achievement at T2	0.00	1.00	−5.24	1.39	−0.11	0.00	−0.07	−0.05	−0.03	0.39^**^	0.35^**^

*N = 249. Age was expressed by years; academic achievement and family SES were indicated by standardized z-scores*.

* *p < 0.05*;

** *p < 0.01*.

### Mediation Analyses

**Figure 2:**
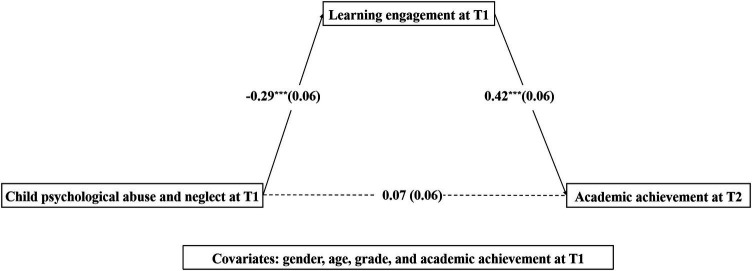
The mediation effect of learning engagement at T1 in the relationship between child psychological abuse and neglect at T1 and children’s academic achievement at T2.

First, the total effect of CPAN at T1 on children’s academic achievement at T2 was statistically nonsignificant (total effect size = −0.05, *SE* = 0.07, *t* = −0.72, *p* = 0.41, bootstrapped 95% CI = [−0.18, 0.08], *R*^2^ = 0.10) when gender, age, grade, and academic achievement at T1 were under control. Then, the results (Figure 2) indicated that CPAN at T1 was negatively associated with learning engagement at T1 (*β* = −0.29, *SE* = 0.06, *p* < 0.001), which in turn positively predicted academic achievement at T2 (*β* = 0.42, *SE* = 0.06, *p* < 0.001). The bootstrapped 95% CI confirmed that the mediation of learning engagement at T1 was significant (*β* = −0.12, *SE* = 0.05, bootstrapped 95% CI = [−0.23, −0.05]). The results suggested that learning engagement at T1 mediated the relationship between CPAN at T1 and children’s academic achievement at T2, accounting for 63.16% of the total effects. And in our study, *R*^2^ effect-size measures for mediation analysis are 0.05.

Table 2 demonstrated the results of the moderated mediation analysis (Edwards and Lambert, 2007), suggesting that the interaction between learning engagement at T1 and family SES at T1 significantly and negatively predicted children’s academic achievement at T2 (*β* = −0.08, *SE* = 0.03, *p* < 0.05). Thus, Family SES at T1 moderated the relationship between learning engagement at T1 and children’s academic achievement at T2. Table 3 showed the bootstrapping estimates and slope coefficients for the conditional indirect effects of the models. Learning engagement at T1 was stronger correlated to children’s academic achievement at T2 of low family SES. The simple slope tests revealed that the effect of learning engagement at T1 on academic achievement at T2 was stronger for children from low family SES (*β* = 0.45, *t* = 6.22, *p* < 0.001) than those from high family SES (*β* = 0.23, *t* = 2.75, *p* < 0.001).

**Table 2:** The moderated mediation models.

Predictor	Learning engagement at T1			Academic achievement at T2		
	*β*	*SE*	Bootstrapped 95% CI	*β*	*SE*	Bootstrapped 95% CI
Gender	−0.12	0.12	[−0.36, 0.13]	−0.16	0.11	[−0.38, 0.05]
Age	0.06	0.13	[−0.20, 0.32]	−0.12	0.12	[−0.36, 0.11]
Grade	0.04	0.12	[−0.19, −0.28]	0.07	0.10	[−0.13, 0.28]
CPAN at T1	−0.27^***^	0.06	[−0.40, −0.15]	0.10	0.06	[−0.02, 0.22]
Family SES at T1				0.19^***^	0.03	[0.12, 0.25]
Learning engagement at T1 × Family SES at T1				−0.08^*^	0.03	[−0.15, −0.01]
Learning engagement at T1				0.38^***^	0.06	[0.27, 0.50]
*R^2^*	0.11			0.30		
*F*	4.00^***^			11.25^***^		

*N = 249. The models control for gender, age, grade, and academic achievement at T1*.

* *p < 0.05*; ^**^p < 0.01;

*** *p < 0.001*.

**Table 3:** Bootstrap estimates of indirect effects at −1SD and +1SD family SES levels.

SD level	Indirect effect (*β*, *Boot SE*)	Bootstrapped 95% CI
−1SD	−0.15, 0.06	[−0.29, −0.05]
+1SD	−0.06, 0.05	[−0.21, −0.01]

*The results control for gender, age, grade, and academic achievement at T1*.

## Discussion

This study investigated the mechanisms underlying the relationship between CPAN at T1 and children’s academic achievement at T2 when individual factors (i.e., learning engagement) and family factors (i.e., family SES) were considered from a longitudinal perspective. The findings showed that children’s learning engagement at T1 mediated the relationship between CPAN at T1 and children’s academic achievement at T2. Moreover, family SES at T1 moderated the pathway from children’s learning engagement at T1 to academic achievement at T2. Specifically, the relationship between children’s learning engagement and academic achievement was stronger for those from low SES families.

### Child Abuse and Neglect and Academic Achievement

Our findings showed that the relationship between CPAN at T1 and academic achievement at T2 was not significant when gender, age, grade, and academic achievement at T1 were under control, which was inconsistent with our hypothesis. There might be two possible explanations. First, this might be related to the broad conceptual implications of CPAN. Previous studies have shown that neglect is more strongly correlated to academic deficits than other forms of abuse (Gauthier et al., 1996; Fantuzzo et al., 2011; Romano et al., 2015). For example, Maclean et al. (2016) found that children’s poorer academic achievement was strongly correlated with neglect rather than emotional abuse. Researchers believed that neglect was more detrimental to academic achievement because children’s basic needs were chronically unmet. These children may not have access to the resources needed for early development, including the resources they need to succeed in school (Fantuzzo et al., 2011). As a result, children who suffer from chronic neglect struggle to reach significant developmental milestones in areas related to academic achievement (McGuire and Jackson, 2018). However, CPAN, in our study, included not only neglect but also multiple forms of abuse such as verbal threats and humiliation of children, which may have prevented us from finding a direct link between CPAN and academic achievement. Future research could expand the sample size and make a clear distinction between neglect and abuse to examine how different forms of CPAN correlated to children’s academic achievement. Second, the correlation between variables may become insignificant when additional variables are more tightly controlled (Boden et al., 2007; Amland et al., 2020; Xiong et al., 2021). Therefore, no direct association between CPAN and academic achievement was found in this study.

### Mediation of Children’s Learning Engagement

The results showed that learning engagement at T1 fully mediated the relationship between CPAN at T1 and academic achievement at T2, which supported hypothesis 2 and was consistent with Mullins and Panlilio (2021). This indirect effect can be accounted for as follows. Firstly, children who suffered psychological abuse and neglect find it difficult to connect to teachers and peers (Bigras et al., 2015; He et al., 2015), and they lack a sense of belonging to school (Sperry and Widom, 2013), reducing emotional learning engagement. Secondly, children with CPAN could not engage constructively in learning activities (Furrer and Skinner, 2003), which impedes behavioral learning engagement. Next, abused and neglected children were chronically exposed to an unsafe and threatening environment, so they find it difficult to focus their attention and make efforts to complete learning tasks. (Van Harmelen et al., 2014; Hawkins et al., 2021). In other words, they lack cognitive engagement in learning activities. Based on these, exposure to CPAN had a negative impact on children’s learning engagement. Moreover, according to learning motivation theory (Skinner et al., 2009), learning engagement, as a motivational factor, played a critical role in children’s academic achievement. For example, low cognitive engagement of a child would make him unwilling to study hard, which impeded academic achievement (Quílez-Robres et al., 2021).

### Moderation of Family SES

**Figure 3:**
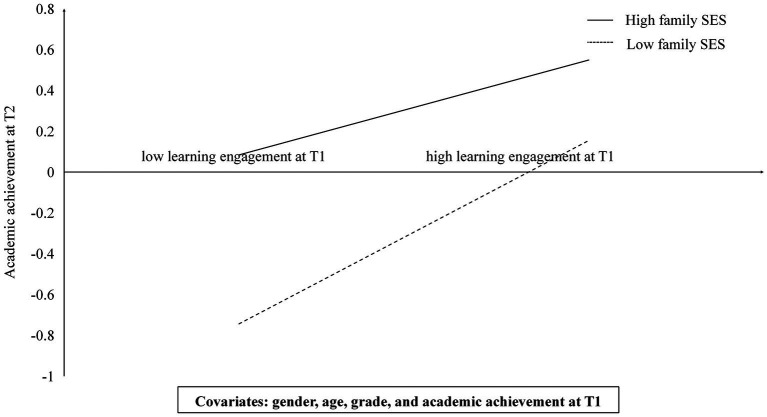
Family SES at T1 as a moderator in the association between learning engagement at T1 and academic achievement at T2. Two levels of family SES are represented graphically: a standard deviation above and below the mean.

The hypothesis that family SES at T1 moderated the path from learning engagement at T1 to academic achievement at T2 was confirmed in this study. Specifically, the relationship between learning engagement at T1 and academic achievement at T2 was stronger for children from low SES families. This phenomenon could be explained by the “compensation effects” (Wang et al., 2017). Children from higher SES families had more learning resources and support conducive to learning achievement (Shi and Tan, 2021). However, since children from low SES families have limited learning resources, learning motivation behavior (i.e., learning engagement) compensates for this deficiency (Shah et al., 2018). Thus, learning engagement had a greater impact on the children from low SES families. It is worth noting that among all the participants, children with high family SES at T1 and learning engagement at T1 got the best academic achievement at T2. Children with lower family SES and learning engagement at T1 got the worst (Figure 3). These findings could be accounted for by previous studies (Poon, 2020), low family SES and low learning engagement were could be dual risk factors for academic achievement of children, showing “compensatory effect” is limited and cannot fully compensate for the academic risks caused by low family SES (Wang et al., 2017). Therefore, educators should pay more attention to children with lower family SES who has lower learning engagement. This moderation further supported that more learning engagement could compensate for the lower academic achievement of the children from lower SES families.

### Limitations and Future Directions

Firstly, previous research had shown that the relationship between CPAN and children’s academic achievement varied according to CPAN forms (Coohey et al., 2011; Maclean et al., 2016). However, these different forms of CPAN were not distinguished in the study. It is still unknown whether these differences affect the moderated mediation model. CPAN in the study was also limited within the family. Future studies could refine instruments to further distinguish and compare different forms or scales of abuse (e.g., physical versus mental abuse and neglect; in-home versus out-of-home abuse and neglect) to show different effects in the moderated mediation model. Secondly, the participants in this study were mainly from one primary school in a medium-sized city in China, which cannot represent the scope of family SES in China. According to the challenge model (Fergus and Zimmerman, 2005), the relationship between family risk factors (i.e., SES) and children’s academic outcomes (i.e., academic achievement) is nonlinear. Future studies could include subjects from extreme family SES and explore whether its moderation on the pathway from learning engagement to academic achievement differed. Besides, our participants also include a group of young children. Although we have taken a lot of measures to promote students’ understanding, including having a strict and uniform application process, offering detailed explanation before testing and providing one-to-one explanation to unclear students, there was a possibility of information bias among younger children. In the future, students in fourth grade and above should be included for further verification. Thirdly, CPAN and learning engagement in this study both relied on children’s self-reports, which may to some bias (Hambrick et al., 2014). Future studies could validate the results by using objective measures based on different samples (e.g., teachers and parents). Finally, the following up time was a semester (6 months) in the current study. It is more convincing to infer causality between early CPAN and children’s academic achievement with a longer time interval in a follow-up design. Besides, the study was conducted during the COVID-19 pandemic, so results in a normal situation are needed.

### Conclusion and Educational Implications

This longitudinal study demonstrated how individual factors (i.e., learning engagement) and family factors (i.e., family SES) affect the relationship between CPAN and children’s academic achievement. Specifically, learning engagement at T1 mediated the correlation between CPAN at T1 and academic achievement at T2. Family SES at T1 moderated the pathway from learning engagement at T1 to children’s academic achievement at T2.

The findings provided guidance for the government, schools, and parents. Firstly, the government should provide more policy support for children exposed to CPAN and from low SES families because they are more prone to have poor academic achievement (Dube and McGiboney, 2018). Secondly, schools and teachers should focus more on the learning engagement of children of CPAN to mitigate the negative impact on academic achievement. For example, they can improve children’s cognitive engagement through attention training, emotional engagement by improving the teacher-student relationship, and behavioral engagement by measures such as raising their rule awareness. Thirdly, parents from low SES families should focus more on their children’s learning engagement. For instance, mothers can provide more emotional warmth and fathers can provide more behavioral guidance to improve children’s learning engagement (Pereira et al., 2016; Wang et al., 2019).

## Data Availability Statement

The raw data supporting the conclusions of this article will be made available by the authors, without undue reservation.

## Ethics Statement

The studies involving human participants were reviewed and approved by the Ethics Committee of the Department of Psychology, School of Humanities and Social Sciences, Beijing Forestry University. Written informed consent to participate in this study was provided by the participants’ legal guardian/next of kin.

## Author Contributions

XY, JL, and ZL contributed to the conception and design of the study. XY and HL organized the database. XY is the gatekeeper of the article as a whole. JL and ZL performed the statistical analysis and wrote the first draft of the manuscript. JY and XL wrote parts of the manuscript. All authors contributed to the article and approved the submitted version.

## Funding

This research was supported by the Medium- and Long-term Research Projects for Teachers, Beijing Forestry University (BJFU2021ZCQ01) to XL.

## Conflict of Interest

The authors declare that the research was conducted in the absence of any commercial or financial relationships that could be construed as a potential conflict of interest.

## Publisher’s Note

All claims expressed in this article are solely those of the authors and do not necessarily represent those of their affiliated organizations, or those of the publisher, the editors and the reviewers. Any product that may be evaluated in this article, or claim that may be made by its manufacturer, is not guaranteed or endorsed by the publisher.

## Appendix 1

The subgroup (sensitivity) analysis among the fourth graders was conducted. The results (see the table below) indicated that CPAN at T1 was negatively associated with learning engagement at T1 (*β* = −0.27, *SE* = 0.06, *p* < 0.001), which in turn positively predicted academic achievement at T2 (*β* = 0.40, *SE* = 0.09, *p* < 0.001). This indicated significant mediating role of learning engagement at T1. However, family SES at T1 did not moderate the relationship between learning engagement at T1 and children’s academic achievement at T2. We speculate that the insignificant moderating results may due to limited sample (*N* = 99). In the future, we could expand the sample size for fourth graders and above to investigate the moderating role of family SES at T1.

The moderated mediation models in fourth graders.

**Table:**

Predictor	Learning engagement at T1			Academic achievement at T2		
	*β*	*SE*	Bootstrapped 95% CI	*β*	*SE*	Bootstrapped 95% CI
Gender	0.18	0.21	[−0.23, 0.60]	0.37^*^	0.18	[0.03, 0.72]
Age	−0.31	0.30	[−0.91, 0.29]	−0.23	0.25	[−0.72, 0.27]
CPAN at T1	−0.27^***^	0.06	[−0.45, −0.09]	0.06	0.08	[−0.10, 0.22]
Family SES at T1				0.12^*^	0.05	[0.12, 0.25]
Learning engagement at T1 × Family SES at T1				−0.04	0.05	[−0.14, 0.05]
Learning engagement at T1				0.40^***^	0.09	[0.23, 0.57]
*R^2^*	0.11			0.31		
*F*	4.00^***^			5.84^***^		

*N = 99. The models control for gender, age, grade, and academic achievement at T1*.

* *p < 0.05*; ^**^*p < 0.01*;

*** *p < 0.001*.
